# Risk Factors for the First Episode of *Klebsiella pneumoniae* Resistant to Carbapenems Infection in Critically Ill Patients: A Prospective Study

**DOI:** 10.1155/2013/850547

**Published:** 2013-12-18

**Authors:** Konstantinos Mantzarlis, Demosthenes Makris, Efstratios Manoulakas, Marios Karvouniaris, Epaminondas Zakynthinos

**Affiliations:** Department of Critical Care, School of Medicine, University of Thessaly, University Hospital of Larissa, 41110 Thessaly, Greece

## Abstract

*Objective*. To identify risk factors for the first episode of *Klebsiella Pneumonia * resistant to carbapenems (KPRC) infection in critically ill patients. *Design, Setting, and Methods.* This prospective cohort study was conducted in a 12-bed general Intensive Care Unit (ICU) in a University Hospital on ICU patients who required mechanical ventilation (MV) for >48 hours during a 12-month period. Clinical and microbiologic data were studied. Characteristics of KPRC patients were compared with those of critically ill patients who presented nonmultidrug resistant (MDR) bacterial infections or no documented infection at all. *Results*. Twenty-five patients presented KPRC infection, 18 presented non-MDR bacterial infection, and 39 patients presented no infection. Compared to patients without documented infection or infected by non MDR bacteria, patients with KPRC infection had received more frequently or for longer duration antibiotics against Gram-negative bacteria (carbapenems, colistin *P* < 0.05). Duration of colistin administration prior to KPRC isolation was independently associated with increased frequency of KPRC infection (odds ratio, 1.156 per day; 95% confidence interval, 1.010 to 1.312; *P* = 0.025). KPRC patients stayed longer in the ICU and received mechanical ventilation and sedation for longer periods and presented increased mortality (*P* < 0.05). *Conclusion*. KPRC infection is an emerging problem which might be more common in patients with previous use of antibiotics and especially colistin.

## 1. Introduction

The management of multidrug resistant (MDR) bacterial infections in the Intensive Care Unit (ICU) is a challenging issue for both physicians and infection control teams. In the United States, Gram-negative bacteria (GNB) account for about 70% of these types of infections in the ICU setting [1] and are associated with significant morbidity and mortality [2]. At the same time, GNB resistance to antibiotics is growing over the years and has become an emerging concern in critical care [3] since the antimicrobial options are very restricted [4].

*Klebsiella pneumoniae* resistant to carbapenems (KPRC) infection is one of the most threatening GNB [5] and unfortunately is rapidly spreading between countries [6–11]. Despite these challenges, there is only a few evidence-based data regarding the risk factors associated with increased frequency of KPRC infection.

In the present prospective study, we aimed to identify possible risk factors for KPRC infection in the critical care setting. These assessments might be crucial in implementing efficient infection control measures to limit the spread of these pathogens and to develop effective strategies for prevention.

## 2. Patients and Methods

This prospective cohort study was conducted in a 12-bed ICU in the University Hospital of Larissa, Thessaly, during a 12-month period, between 2011 and 2012. Inclusion criteria were (a) admission in the ICU for medical or surgical causes and (b) intubation and mechanical ventilation for >48 hours. Exclusion criteria were (a) age <18 years old, (b) pregnancy, (c) ICU readmission, (c) other MDR infections, and (d) multiple bacterial infections. The first episode of KPRC infection was accounted. Patients with KPRC infection were compared with patients who were ventilated >48 h and presented either non-MDR bacterial infection or no infection at all. Institutional review board approved the study that was conducted in accordance with current institutional regulations.

### 2.1. Outcome

Identification of risk factors for the first episode of KPRC infection in ICU was the primary outcome in this study.

### 2.2. Definitions

KPRC was defined according to the revised Minimum Inhibitory Concentration (MIC) breakpoints as previously described [12]. KPRC infection was defined as clinical manifestation of infection which was microbiologically confirmed by isolation of KPRC in cultured material. Definition of ventilator associated pneumonia (VAP) included the presence of new or progressive radiographic infiltrate associated with two of the three following criteria: (1) temperature >38.5°C or <36.5°C, (2) leukocyte count >10000/*μ*L or <1500/*μ*L, and (3) purulent tracheal aspirate. In addition, a positive tracheal aspirate in quantitative cultures (≥10^5^ cfu/mL) or a positive bronchoalveolar lavage culture (≥10^4^ cfu/mL) was required to confirm the diagnosis. Blood stream infection and catheter associated urinary tract infection were defined according to Center of Disease Control (CDC) criteria [13, 14]. Isolation of KPRC in biological samples without criteria for clinical infection was considered as colonization. We considered as immunocompromised any patient who was transplanted or received immunosuppressive agents, including corticosteroids. Tracheal aspirate or other type of cultures were quantitative except blood cultures. We considered non-MDR bacteria strains susceptible to the next three antibiotic categories: third generation cephalosporins, antipseudomonal penicillins and quinolones [15]. Previous hospitalization was defined as admission to hospital or other health care facilities for >48 h during the last three months. Appropriate empirical antimicrobial treatment referred to administration at least one of in vitro active antimicrobials against the study isolates within 24 hrs from infection onset. Appropriate definitive treatment was considered as the administration of in vitro active antibiotics for at least 48 hrs [16].

### 2.3. Clinical Assessment

For all study patients, the following characteristics were prospectively recorded: age, sex, illness severity based on Acute Physiology and Chronic Health Evaluation Score II (APACHE II), Sequential Organ Failure Assessment (SOFA) score at admission, type of admission (transfer to the ICU from a ward/emergency department), history of hospitalization during the last 3 months before current admission, history of invasive procedures (gastroscopy, colonoscopy, or bronchoscopy) or surgery, medical history, history of antibiotic use and type and duration of antibiotics used, and corticosteroid treatment during ICU stay. For survivors and nonsurvivors, several characteristics that might affect mortality were recorded: age, sex, severity of illness, comorbidities, invasive procedures, appropriate antibiotic treatment, total duration of mechanical ventilation (MV) and sedation, and KPRC infection.

### 2.4. Microbiology

Identification and susceptibility testing of *K. pneumoniae* blood isolates were performed by the Vitek 2 automated system (bioMerieux, Marcy l' Etoile, France). Phenotypic screening for carbapenemases was performed for all isolates exhibiting reduced susceptibility to carbapenems (MIC > 1 *μ*g/mL) by using the modified Hodge test and the combined disk tests using carbapenems with and without EDTA or boronic acid [17, 18].

### 2.5. Management of Infection and Infection Control Policy

Nature and duration of treatment for each infection were at physicians' discretion. Infection control policy included isolation techniques in patients with MDR bacteria and continuous surveillance of nosocomial infections. Sedation and ventilator weaning procedures were standard throughout the whole study period; the common policy for sedation in our unit includes mainly remifentanil/propofol/midazolam, and sedation regimens were at physicians' discretion throughout the study. Our unit practices the respiratory bundle suggested by the CDC [19] unless contraindicated. Oral decontamination was performed daily with the use of chlorhexidine, while prophylactic local antibiotics as part of Selective Oropharyngeal or Selective Digestive Decontamination (SDD) was not applied. Surveillance cultures (nasal, oral, rectal, and tracheal aspirate) were performed routinely on admission and once weekly. Blood counts and biochemical measurements were performed daily.

### 2.6. Statistical Analysis

Results are presented as frequency (%) for qualitative variables or median (25th and 75th quartiles) for quantitative variables. Normality of data distribution was assessed by Kolmogorov Smirnov test. Qualitative variables were compared using Chi-square test or Fisher's exact test where appropriate; quantitative variables were compared by one way ANOVA (Bonferroni post hoc test). Multivariate analyses were performed to determine variables associated with KPRC infection or mortality. Only those variables which were significantly associated with KPRC infection or mortality in the univariate analysis were entered in the stepwise logistic regression models. For KPRC infection, analysis was performed between two groups (patients with KPRC and all other patients); colistin duration, MV, invasive procedures were assessed as risk factors for KPRC infection. Exposure to potential risk factors was taken into account only before diagnosis of KPRC infection. Patients with KPRC colonization were not included in analysis. Mortality analysis was performed between two groups (survivors and nonsurvivors); age, invasive procedures, total duration of sedation and MV, medical cause of admission, and KPRC infection were assessed as risk factors. SPSS software (SPSS 17.0, Chicago, IL) was used for data analysis.

## 3. Results 

**Figure 1:**
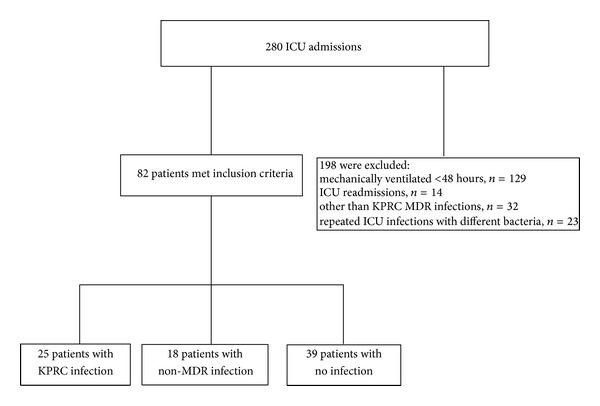
Flow chart of the study.

A total of 280 patients were studied (Figure 1). There were 25 (8.9%) cases of KPRC infection which included 16 (65.4%) bloodstream infections, 6 (23%) respiratory infections, 2 (7.7%) urinary tract infections, and 1 (3.8%) central nervous system (CNS) infection. The 18 non-MDR bacterial infection cases included 2 cases with *Escherichia coli, *5 cases with* Methicilline Sensitive Staphylococcus aureus*, 1 case with* Staphylococcus epidermidis*, 1 case with *Streptococcus pneumoniae, *2 cases with* Klebsiella pneumonia, *2 cases with *Enterococcus faecalis, 2* cases with* Pseudomonas aeruginosa, *1 case with* Enterobacter cloacae, *1 case with* Proteus mirabilis*, and 1 case with* Serratia marcescens. *KPRC was isolated at mean (SE) 13.1 (1.9) ICU days and non-MDR bacteria at 6.78 (1.756) ICU days. From the other 55 patients who presented infections with MDR pathogens other than KPRC, 32 were infected with *Acinetobacter baumannii *and the rest had multibacterial infections. The percentage of the uninfected patients which were colonized with bacteria in the study period was estimated up to 39.5%; the percentage of uninfected patients with KPRC colonization was up to 6.9%. Characteristics of participants are presented in Tables 1–3.

### 3.1. Risk Factors for KPRC Infection

Baseline characteristics between groups are not significantly different and are presented in Table 1. Prior use and the duration of administration of specific antibiotics, the duration of MV, and the performance of invasive procedures were significantly associated with KPRC infection (Tables 2 and 3). Patients with KPRC infection received carbapenems and colistin for longer duration and carbapenems more frequently (odds ratio (OR) 95%; confidence interval (CI) 5.42 (1.25–23.49)) prior to infection compared to patients who presented non-MDR infection (Table 3). Patients with KPRC infection received colistin more frequently (4.24 (1.43–12.56)) and for longer period (days) compared to patients who presented no documented infection (Table 3). In addition, KPRC patients had received MV for longer periods before pathogen isolation compared to the other two groups, and they underwent more frequently invasive procedures (Table 2). Inappropriate empirical treatment was associated more frequently with KPRC infection (Table 3).

Multivariate analysis revealed that colistin duration of administration was an independent risk factor for KPRC infection (OR (95%CI) 1.15 (1.02–1.31) per day of administration) (*P* = 0.025).

### 3.2. Mortality and Morbidity Indices in Patients with KPRC Infection

KPRC patients in comparison with patients who presented no documented infection had longer ICU stay, mortality (4.35 (1.5–12.7)), and total duration of mechanical ventilation and sedation (Table 4). Survivors compared to non-survivors had younger age (Table 5), received MV or sedation for shorter duration and underwent less frequently invasive procedures (0.16 (0.03–0.8)) while they were more likely admitted due to surgical and nonmedical causes (0.33 (0.13–0.8)) and presented less frequently KPRC infection (3.25 (1.2–8.6)).

Multivariate analysis showed that age (years) (1.048 (1.006–1.093))  (*P* = 0.023) and immunodeficiency (25.22 (1.94–327.19))  (*P* = 0.014) were independent risk factors for ICU mortality.

## 4. Discussion

In the present study, we aimed to identify clinical risk factors for the first episode of KPRC infection in ICU. Our findings suggest that KPRC infection was associated with the prior use of antibiotics, in particular carbapenems and most notably with colistin. To our knowledge the association between KPRC and the use of colistin has not yet been reported. KPRC was also associated with the performance of invasive procedures and the duration of mechanical ventilation prior to infection. In this respect, our study suggests that infection control policies should reinforce measures to prevent excessive antibiotic use and to be cautious with the use of colistin.

Data regarding risk factors for KPRC infection in the ICU are limited. Most data have been derived from mixed medical and critical care populations; the prevalence of critically ill patients among those studies is unclear. Moreover, most previous studies were retrospective; this may insert bias in the interpretation of the results, especially in the diagnosis of KPRC infection, for example, differentiation between infection and colonization. In this respect, definitive conclusion for the risk factors of KPRC infections in the critical care setting is hard to be drawn from available evidence. To our knowledge, the present study is the first prospective study which aimed to identify risk factors for KPRC infection in the ICU setting and may provide useful data in the implementation of effective infection control policies.

We found an association between KPRC infection and the previous use of antibiotics. A relationship between prior carbapenem use and KPRC infection might be expected [20–24]. Carbapenems can destroy the susceptible proportion of strains which is part of patient's colonies, so opportunistic infection could be accomplished by the resistant one. The results of the present study are consistent with our retrospectively collected data from our centre reported previously [25] where KPRC patients had also received more often (87% versus 40% *P* = 0.003) and for longer periods (days) (8.8 (1.8) versus 1.75 (0.6) *P* < 0.001) carbapenems.

Moreover, we found that colistin was also associated with increased risk of KPRC infection and that the duration of colistin administration was an independent risk factor for KPRC infection. To our knowledge this association has not yet been reported. Colistin is an old antibiotic and potentially toxic to kidneys that has been used increasingly during the last years due to the emergence of Gram-negative MDR bacteria. The association between colistin and KPRC might illustrate the “cul-de-sac” we are in. The emergence of MDR Gram-negative infection requires treatment with an old antibiotic such as colistin, but the use of colistin may promote the selection of “super bugs” such as KPRC. It is therefore likely that the increasing use of antibiotics with a broad spectrum against GNB might progressively modify the microbiological flora of critically ill patients in favour of KPRC and of other “superbugs.” Additionally, little is known about the pharmacokinetics/dynamics of colistin [26, 27]. Notably, colistin administration as the appropriate treatment—based on in vitro bacterial susceptibility to antibiotics—was not associated with improved survival in KPRC infection according to previous studies [20].

In light of our findings, one should be cautious with the empirical and prolonged use of colistin, especially when other potential factors for KPRC infection are present. Furthermore, our study questions the usefulness of SDD policies which include colistin, in ICU settings where KPRC is prevalent. In our unit we do not use decontamination strategies with colistin and we therefore cannot provide data in this issue. Nevertheless, we advocate in favour of appropriate de-escalation strategies that should be considered when culture results are available. Studies about the optimum duration of colistin administration and the implementation of Antimicrobial Stewardship Programs may improve antimicrobial use in Intensive Care Units in order to prevail growing antimicrobial resistance. Certainly, fast and accurate diagnostic tests for GNB and more specifically for KPRC infection would be also very helpful in the de-escalation of treatment, but to our knowledge, tests for KPRC are not available at the moment.

In the present investigation, there was also an association between KPRC infection and the duration of MV. Mechanical ventilation is a treatment option that is often necessary in critical illness and it is related with the burden of the disease; its duration might be considered as a marker of disease severity [28]. Previous studies have underlined the association between KPRC infection and the burden of critical illness [29, 30]. Moreover, the application of MV includes procedures that interrupt the physiologic defence barriers and usually requires sedation, and thus it might favour the development of nosocomial infections [31].

The fact that KPRC was also associated with the performance of invasive procedures, with sedation and with prolonged ICU stay, is in agreement with the above hypothesis. Longer hospitalisation may additionally predispose in greater risk for colonization with KPRC strains [32, 33] through patient-to-patient transmission. Unfortunately, the present study has not examined mechanisms of colonization with KPRC strains which could be the scope of a future study.

In the present study, patients who presented KPRC infection in the ICU had increased mortality, required longer duration of mechanical ventilation and sedation, and stayed for a longer period in the ICU compared to patients who had not acquired an infection in the ICU. Recent retrospective reports provided also evidence that KPRC infection might be significantly associated with adverse outcomes [20, 25, 29, 34, 35]. In our prospective study, despite that the groups of patients were comparable in terms of age, burden of disease (APACHE), and cause of admission, the number of outcomes was small to draw definitive conclusions. We have to underline that KPRC infection, although associated with ICU mortality in univariate analysis, was not an independent factor for ICU mortality as multivariate analysis revealed. A plausible explanation for this might be that the number of KPRC infected patients included in the study was relatively small; in addition, ICU mortality is multifactorial, and several other factors which have not been assessed in the present study have also an impact on mortality, and they might have obscured a potential association between KPRC and mortality.

In conclusion, our findings provide evidence that prior use of antibiotics, in particular, prolonged use of colistin, is an independent factor for KPRC infection. In this respect, the present study suggests that judicious use of antibiotics against Gram-negative, such as colistin, should be part of the algorithm required to restrict the spread of KPRC infection in the ICU.

## Figures and Tables

**Table 1:** Baseline characteristics of participants.

	KPRC (*N* = 25)	P*	Non-MDR bacterial infection (*N* = 18)	*P* ^#^	No bacteria group (*N* = 39)	*P* ^#^
Sex (male)	14 (56)	0.328	8 (44.4)	0.199	26 (66.7)	0.436
Age (years)	62 (50, 69)	0.150	47 (32, 62)	0.229	62 (40, 76)	0.245
Medical patients	14 (56)	0.597	8 (44)	0.223	17 (43.6)	0.443
Diagnosis during admission						
Sepsis	3 (12)	0.590	3 (17)	0.683	3 (8)	0.671
Neurological disease	4 (16)	0.201	1 (5)	0.127	6 (15)	1.0
Pancreatitis	2 (8)	0.223	0 (0)	1.0	0 (0)	0.149
ARDS	4 (16)	0.277	1 (6)	0.380	2 (5)	0.199
Neurosurgical disease	5 (20)	0.587	4 (22)	1.0	12 (31)	0.397
Abdominal surgery	3 (12)	0.773	1 (5)	0.628	4 (10)	1.0
Trauma patients	2 (8)	0.656	3 (17)	0.634	4 (10)	1.0
Other	2 (8)	0.225	5 (28)	0.110	8 (21)	0.292
APACHE II score	15 (12, 20)	0.204	13 (10, 19)	0.886	15 (10, 18)	0.230
SOFA score	7 (4, 8)	0.768	7 (6, 9)	1.0	7 (5, 9)	1.0
Hospitalization in the last 3 months	6 (24)	0.157	1 (5.6)	0.209	4 (10.3)	0.170
Admission from emergency department	15 (60)	0.355	7 (38.9)	0.223	18 (46.2)	0.315
Diabetes mellitus	5 (20.8)	0.156	2 (11.1)	0.679	2 (5.1)	0.095
Chronic lung disease	2 (8.3)	0.665	3 (16.7)	0.636	6 (15.4)	0.699
Chronic heart disease	7 (29.2)	0.570	4 (22.2)	0.731	14 (35.9)	0.784
Chronic renal failure	2 (8.3)	0.320	0 (0)	0.498	1 (2.6)	0.552
Neurological disease	9 (37.5)	0.108	4 (22.2)	0.333	20 (51.3)	0.311
Chronic liver disease	2 (8.3)	0.585	1 (5.6)	1.0	1 (92.6)	0.552
Malignancy	2 (8.3)	0.401	2 (11.1)	1.0	1 (2.6)	0.552
Immunodeficiency	2 (8.3)	0.940	1 (5.6)	1.0	3 (7.7)	1.0

Data are presented as median (25% and 75% quartiles) or *n* (%); KPRC: *Klebsiella pneumonia *resistant to carbapenems; MDR: multidrug resistant bacteria; APACHE: Acute Physiology and Chronic Health Evaluation; SOFA: Sequential Organ Failure Assessment; ARDS: Acute respiratory distress syndrome; P*: comparison between three groups; *P* ^#^: KPRC versus non-MDR group, KPRC versus no bacteria group. Results are by univariate analysis.

**Table 2:** Clinical characteristics of participants in the ICU before KPRC or non-MDR infection.

	KPRC (*N* = 25)	P*	Non MDR bacterial infection (*N* = 18)	*P* ^#^	No bacteria group (*N* = 39)	*P* ^#^
MV duration (days)	10 (5, 19)	0.003	5 (2, 8)	0.005	7 (4, 11)	0.021
Surgical operation	15 (60)	0.852	12 (66.7)	0.735	23 (59)	1.0
Invasive procedures	6 (24)	0.044	1 (5.6)	0.209	2 (5.1)	0.048
Catheterization of urinary bladder prior ICU admission	1 (4)	0.850	1 (5.6)	1.0	1 (2.6)	1.0
Tracheotomy	7 (28)	0.403	2 (11.1)	0.283	8 (20.5)	1.0
Sedation	22 (88)	0.252	18 (100)	0.252	37 (95)	0.371
CVVHDF use	4 (16)	0.781	2 (11.1)	1.0	4 (10.3)	0.701
CVVHDF duration (days)	0 (0, 0)	0.179	0 (0, 0)	0.396	0 (0, 0)	0.271
Corticosteroids (mg of hydrocortisone/day)	0 (0, 1)	0.936	0 (0, 0)	1.0	0 (0, 0)	1.0

Data are presented as median (25% and 75% quartiles) or *n* (%); KPRC: *Klebsiella pneumonia *resistant to carbapenems; MDR: multidrug resistant bacteria; MV: mechanical ventilation; ICU: Intensive Care Unit; CVVHDF: Continuous veno-venous hemodiafiltration; invasive procedures, gastroscopy, colonoscopy, or bronchoscopy; P*: comparison between three groups; *P* ^#^: KPRC versus non-MDR group, KPRC versus no bacteria group. Results are by univariate analysis.

**Table 3:** Antibiotic treatment administered to participants.

	KPRC (*N* = 25)	P*	Non-MDR bacterial infection (*N* = 18)	*P* ^#^	No bacteria group (*N* = 39)	*P* ^#^
Antibiotics in the last 3 months	5 (20)	0.336	2 (11.1)	0.680	3 (7.7)	0.245
Antibiotics during hospitalization prior to infection	24 (96)	0.022	14 (77.8)	0.144	38 (97.4)	1.0
Use of carbapenems	13 (52)	0.062	3 (16.7)	0.026	15 (38.5)	0.313
Duration of carbapenem use (days)	3 (0, 11)	0.025	0 (0, 0)	0.021	0 (0, 6)	0.409
Use of antipseudomonal penicillins	8 (32)	0.474	3 (16.7)	0.309	12 (30.8)	1.0
Duration of antipseudomonal penicillins use (days)	0 (0, 4)	0.265	0 (0, 0)	0.330	0 (0, 4)	1.0
Use of quinolones	7 (28)	0.403	2 (11.1)	0.263	8 (20.5)	0.553
Duration of quinolones use (days)	0 (0, 4)	0.154	0 (0, 0)	0.162	0 (0, 0)	1.0
Use of cephalosporins 3rd generation	5 (20)	0.217	6 (33.3)	0.480	16 (41)	0.105
Duration of cephalosporins 3rd generation use (days)	0 (0, 0)	0.886	0 (0, 2)	1.0	0 (0, 2)	1.0
Use of cephalosporins 4th generation	2 (8)	0.341	0 (0)	0.502	1 (2.6)	0.555
Duration of cephalosporins 4th generation use (days)	0 (0, 0)	0.228	0 (0, 0)	0.423	0 (0, 0)	0.395
Use of colistin	14 (56)	0.028	7 (38.9)	0.358	9 (23.1)	0.015
Duration of colistin use (days)	2 (0, 13)	0.001	0 (0, 4)	0.022	0 (0, 0)	0.001
Use of tygecycline	5 (20)	0.07	2 (11.1)	0.680	1 (2.6)	1.0
Duration of tygecycline use (days)	0 (0, 0)	0.096	0 (0, 0)	0.392	0 (0, 0)	0.108
Use of aminoglycosides	2 (8)	0.934	2 (11.1)	1.0	4 (10.3)	1.0
Duration of aminoglycosides use (days)	0 (0, 0)	0.560	0 (0, 0)	1.0	0 (0, 0)	1.0
Aerosolized colistin	4 (16)	0.673	4 (22)	1.0	5 (13)	1.0
Appropriate empirical antibiotic treatment	15 (60)	0.38	16 (88)	0.046	NA	NA
Appropriate definitive antibiotic treatment	23 (92)	0.229	18 (100)	0.502	NA	NA

Data are presented as median (25% and 75% quartiles) or *n* (%); KPRC: *Klebsiella pneumonia *resistant to carbapenems; MDR: multidrug resistant bacteria; appropriate empirical antimicrobial treatment, administration of in vitro active antimicrobials against the study isolates within 24 h from infection onset; appropriate definitive antibiotic treatment, administration of in vitro active antibiotics for at least 48 h; P*: comparison between three groups; *P* ^#^: KPRC versus non-MDR group, KPRC versus no bacteria group. Results are by univariate analysis.

**Table 4:** Duration of ICU stay, death, mechanical ventilation, and sedation in patients with KPRC, non-MDR infection, and no infection.

	KPRC (*N* = 25)	P*	Non-MDR bacterial infection (*N* = 18)	*P* ^#^	No bacteria group (*N* = 39)	*P* ^#^
ICU duration (days)	24 (15, 32)	<0.001	20 (9, 32)	0.433	9 (6, 13)	<0.001
Death	15 (60)	0.022	8 (44.4)	0.365	10 (25.6)	0.009
MV duration (days)	20 (9, 31)	<0.001	18 (9, 25)	0.435	7 (4, 11)	<0.001
Duration of sedation (days)	8 (2, 18)	0.003	9 (5, 15)	0.967	4 (2, 8)	0.003

Data are presented as median (25% and 75% quartiles) or *n* (%); KPRC: *Klebsiella pneumonia *resistant to carbapenems; MDR: multidrug resistant bacteria; MV: mechanical ventilation; ICU: Intensive Care Unit; P*: comparison between three groups; *P* ^#^: KPRC versus non-MDR group, KPRC versus no bacteria group. Results are by univariate analysis.

**Table 5:** Characteristics of survivors and nonsurvivors in the ICU.

	Survivors (*N* = 49)	Nonsurvivors (*N* = 33)	*P*
Sex (male)	31 (63.3)	23 (69.7)	0.638
Age (years)	55 (36, 37)	62 (52, 73)	0.023
Medical patients	18 (36.7)	21 (63.6)	0.024
APACHE II score	14 (10, 19)	15 (13, 18)	0.710
SOFA score	7 (5–8)	7 (5, 9)	0.121
Diabetes mellitus	3 (6.1)	6 (18.8)	0.144
Chronic lung disease	5 (10.2)	6 (18.8)	0.328
Chronic heart disease	15 (30.6)	10 (31.1)	0.571
Other comorbidities	25 (51)	14 (42)	0.546
Immunodeficiency	1 (2)	5 (15.6)	0.033
Invasive procedures	2 (4.1)	7 (21.2)	0.027
Appropriate empirical antibiotic treatment	14 (70)	17 (73.9)	0.521
Appropriate definitive antibiotic treatment	19 (95)	22 (95.7)	0.720
Total ICU duration (days)	12 (7, 23)	14 (9, 28)	0.230
MV total duration (days)	9 (4, 16)	14 (9–28)	0.007
Sedation total duration (days)	4 (2, 10)	9 (5, 15)	0.009
KPRC infection	10 (20.4)	15 (45.5)	0.027

Data are presented as median (25% and 75% quartiles) or *n* (%); KPRC: *Klebsiella pneumonia *resistant to carbapenems; other comorbidities included hematological disease, chronic kidney and chronic liver disease; appropriate empirical antimicrobial treatment, administration of in vitro active antimicrobials against the study isolates within 24 h from infection onset; appropriate definitive antibiotic treatment, administration of in vitro active antibiotics for at least 48 h; ICU: Intensive Care Unit; APACHE: Acute Physiology and Chronic Health Evaluation; SOFA: Sequential Organ Failure Assessment; MV: mechanical ventilation; Results are by univariate analysis.
